# An Active Lifestyle Reinforces the Effect of a Healthy Diet on Cognitive Function: A Population-Based Longitudinal Study

**DOI:** 10.3390/nu10091297

**Published:** 2018-09-13

**Authors:** Behnaz Shakersain, Debora Rizzuto, Hui-Xin Wang, Gerd Faxén-Irving, Federica Prinelli, Laura Fratiglioni, Weili Xu

**Affiliations:** 1 Aging Research Center, Department of Neurobiology, Care Sciences and Society, Karolinska Institutet/Stockholm University, Widerströmskahuset, plan 10, Tomtebodavägen 18A, 171 65 Solna, Sweden; behnaz.shakersain@ki.se (B.S.); Debora.Rizzuto@ki.se (D.R.); huixin.wang@su.se (H.-X.W.); federica.prinelli@ki.se (F.P.); Laura.Fratiglioni@ki.se (L.F.); 2 Stress Research Institute, Stockholm University, 106 91 Stockholm, Sweden; 3 Division of Clinical Geriatrics, Department of Neurobiology, Care Sciences and Society, Karolinska Institutet, 141 57 Huddinge, Sweden; Gerd.Faxen.Irving@ki.se; 4 Institute of Biomedical Technologies-National Research Council Via Fratelli Cervi 93, 20090 Segrate (MI), Italy; 5 Stockholm Gerontology Research Center, 113 30 Stockholm, Sweden; 6 Department Epidemiology & Biostatistics, School of Public Health, Tianjin Medical University, Tianjin 300070, China

**Keywords:** the Nordic Prudent Dietary Pattern, leisure activities, active life style, cognitive function, population-based cohort study

## Abstract

The joint effect of diet and leisure activity on cognitive function remains unknown. We aimed to verify the hypothesis that an active lifestyle reinforces the effect of the Nordic Prudent Dietary Pattern (NPDP) on cognitive function. A total of 2223 dementia-free Swedish adults aged ≥60 with Mini-Mental State Examination (MMSE) scores ≥27 were followed for an average of 6 years. MMSE was tested during follow-ups. Diet was assessed by food frequency questionnaire. The NPDP index was calculated and tertiled (low, moderate, and high adherence). Participation in physical, mental and social activities was trichotomised (low, moderate, and intense). An active lifestyle was defined based on the participation in each activity. Data were analyzed using mixed-effects models. Moderate-to-high adherence to NPDP was associated with a reduced decline in the MMSE score (β: 0.19, 95% Confidence Interval (CI): 0.14–0.24). This association became stronger when combined with moderate-to-intense physical (β: 0.34, 95% CI: 0.2–0.45), mental (β: 0.29, 95% CI: 0.21–0.37), or social (β: 0.27, 95% CI: 0.19–0.34) activities. An active lifestyle strengthened the effect of NPDP on cognitive function by two times, and further lowered risk of MMSE decline by 30%. Thus, an active lifestyle reinforces the effect of a healthy diet on preserved cognitive function, and further decreases the risk of cognitive decline.

## 1. Introduction

With a rising prevalence of dementia around the world, there is an urgent need to identify opportunities for prevention. Worldwide, 46.8 million people were diagnosed with dementia in 2015 [1]. As up to half of dementia cases may be attributable to modifiable lifestyle-related factors [1], many researchers have attempted to address the effects of lifestyle factors on the risk of developing dementia [2]. Indeed, physical, mental, and social activities have been associated with reduced risk of cognitive impairment and dementia [3,4], in spite of some inconsistencies in previous findings [5]. Healthy diets (such as the Mediterranean diet) have also been shown a beneficial effect on cognitive function in some of the Northern American, Australian and Southern European populations [6,7,8], but not in all [9,10,11].

Lifestyle is a complex set of inter-related behaviors and exposures. It is speculated that individuals with healthier eating habits are, in general, more health-conscious, and tend also to have an active lifestyle. Epidemiological studies suggest that life-time exposures, such as healthy lifestyle factors may increase the cognitive reserve or resilience [2,12]. The interplay between multiple lifestyle factors may have a different effect on underlying neurodegenerative processes in the brain [13]. Although the individual impacts of various lifestyle factors on cognitive impairment and dementia has been repeatedly investigated [13,14], the combined effect of diet and leisure activities on cognitive function remains unclear. The magnitude of the potential joint effect of these lifestyle factors on cognitive function in older individuals is of great interest from a public health perspective.

Within the cohort of a Northern European older population, we recently identified the Nordic Prudent Dietary Pattern (NPDP) as a healthy eating pattern associated with preserved cognitive function [15]. We hypothesized that high adherence to NPDP and an active lifestyle would have joint benefits in decelerating cognitive decline. In the present study, we sought to examine the combined effect of a healthy diet and leisure activities, including physical, mental, and social dimensions, on cognitive decline using the 6-year follow-up data from a population-based cohort of Swedish older adults.

## 2. Materials and Methods

### 2.1. Study Design

The study population was drawn from the Swedish National study on Aging and Care-Kungsholmen (SNAC-K), an ongoing longitudinal study on a random sample of community residents aged ≥60 in Kungsholmen, central Stockholm, Sweden (http://www.snac-k.se). Of the 4590 alive and eligible individuals, 3363 (73.3%) underwent baseline examinations (March 2001–June 2004). The participants consisted of eleven age cohorts. Follow-up examinations had been performed at 6-year intervals for younger age cohorts (60, 66, and 72 years), and at 3-year intervals for older age cohorts (78, 81, 84, 87, 90, 93, 96, and ≥99 years) because of a higher attrition rate among older groups. After exclusion of individuals with dementia (*n* = 311) or missing data on dementia (*n* = 10), people without dementia whose Mini-Mental State Examination (MMSE) scores were <27 (*n* = 306) or whose MMSE score was missing (*n* = 5), and those with more than 20% missing data on the semi-quantitative food frequency questionnaire (SFFQ; *n* = 508), 2223 individuals were left in the current study [15]. These participants, if alive and available, were examined at the first (2004−2007) and the second follow-up (2007−2010) examinations (Figure S1).

SNAC-K was approved by the Regional Ethical Review Board in Stockholm, Sweden, and written informed consent was obtained from each participant, or in case of cognitive impairment, from a close family member at baseline.

### 2.2. Data Collection

At baseline and each follow-up, demographic and health-related information (including medications and dietary supplements intake classified in accordance with the Anatomical Therapeutic Chemical (ATC) classification) was obtained via physician examinations, nurse interviews, and self-administered questionnaires. Information regarding the quantity of type of supplements was not available in this study. Chronic diseases were diagnosed by the examining physician on the basis of medical history, clinical examination, laboratory tests, and current medication use in accordance with the ninth and tenth revisions of the International Classification of Diseases [15].

Educational level was defined as elementary, high school, or university using the reported maximum years of formal schooling. Civil status was defined as married (which included those who were cohabiting), single, or widow/divorced. Smoking status was categorized as never-smokers, former-smokers, or current-smokers. For former and current smokers, a total number of years of smoking was reported (0 was assigned to never smokers in analysis). Body Mass Index (BMI) was calculated as weight divided by height squared using the nurse-measured weights (kg) and heights (m). Genotyping for *APOE* (rs429358) was carried out on blood samples that were taken from each participant at baseline. The Swedish Cause of Death Register at the National Board of Health and Welfare was used to assess survival status by the end of the study follow-up. Details of the assessment of these variables have been described in a previously published study [16].

### 2.3. Dementia Diagnosis and Cognitive Function Test

A validated clinical three-step procedure [17] was applied to identify dementia in accordance with the Diagnostic and Statistical Manual of Mental Disorders criteria (4th Edition). Cognitive function was assessed via MMSE tests for global cognitive evaluation at baseline and at each follow-up. Of the younger cohorts without dementia and with MMSE evaluation at baseline (*n* = 1516), about 88.3% of those alive attended the 6-year follow up (no MMSE missing). Of the older cohorts without dementia (*n* = 707), about 88.0% of those alive attended the 3-year follow-up (MMSE missing = 1), and 90.8% of those alive after the first follow-up attended the 6-year follow-up (MMSE missing = 4). Clinically meaningful cognitive decline was defined as a decline in MMSE score of ≤24 [18].

### 2.4. Assessment of Diet

Usual dietary intakes were assessed at baseline using a validated self-administered 98-item SFFQ [19]. Participants were asked to report their average intake frequency of each food and beverage over the past 12 months. The nine possible answers to each question ranged between never and ≥4 times per day. Portion sizes of staple foods (potatoes, rice, and pasta), meat, and vegetables were estimated by providing color photos of 4 plates with increasing portion sizes in the SFFQ. Standard portion sizes (e.g., the size of an average apple as one portion of fruit) were used for other food items. In the current study, the reported frequencies of intakes were used in all analyses. The food composition database of the National Food Administration was considered to estimate each participant’s total calorie intake using MATs software Version 4.03 (Rudans Lättdata, Västerås, Sweden).

The NPDP index, consisting of 15 food and beverage items, was calculated as previously described [15]. In brief, on the basis of the independent associations between each food group (and their sub-items) and MMSE change over time, NPDP was developed as a better predictor of preserved cognitive function in Nordic older populations compared to other four predefined dietary indices, including the Mediterranean-Intervention for Neurodegenerative Delay (MIND), the Mediterranean Diet (MedDietScore), Dietary Approaches to Stop Hypertension (DASH), and the Baltic Sea Diet (BSD). This dietary index reflects high consumptions of non-root vegetables, apples/pears/peaches, pasta/rice, poultry, fish, vegetable oils (mainly rapeseed oil), tea, and water, light to moderate wine intake, and low consumptions of root vegetables (including potatoes), refined grains/cereals, high-fat dairy products, butter/margarine, sugar/sweets/pastries, and fruit juice.

The following procedure was applied to assess the NPDP index score: (1) The intake of each food component was dichotomized using the calorie-adjusted and standardized sex-specific population-median of food intake (frequency per day) as the cut-off to define low versus high consumption. (2) For the consumption of food items presumed to be healthy, a score of 0 was assigned for intakes below the median, and scores of 1 to 5 were assigned to quintiles of intakes above the median. For the consumption of food components presumed to be less healthy, the scoring was reversed. (3) Wine intake was scored as 1 (>0 to ≤1 drink per day for women, and >0 to ≤2 drinks per day for men) or 0 (all other amounts) [20]. (4) The scores assigned to dietary component intakes were summed to a total score.

Higher index scores indicated greater adherence to the NPDP. The index scores were used as continuous and categorical (tertiled as low, moderate, and high adherence; or dichotomized as moderate-to-high vs. low) variables in the data analyses.

### 2.5. Assessment of Leisure Activities

Information on leisure activities was collected through a self-administered questionnaire with the following question: Have you devoted yourself to the below listed entertainment and cultural activities during the last 12 months? The participant was then asked to write or check the best answers of the 11 predefined leisure time activities and the frequency of participation. Leisure activities included physical, mental, and social pursuits outside work-related activities that were performed at least once a week, and they were rated in agreement with previous studies [21,22].

Physical activity was categorized as (1) “intense” if the study participant reported having intense physical exercise (e.g., brisk walking, jogging, long bike rides, intense gym exercise or other sports) at least once a week; (2) “moderate” if the participant reported doing moderate physical exercise (e.g., walking, short bike rides, light gym exercises, or golf) or other physical activities such as gardening, picking mushrooms/berries, hunting/fishing, home repairs, and car mechanics at least once a week; and (3) “low” if the participant reported performing the aforementioned physical exercise/activities less than once a week [21,22].

Mental activities consisted of reading newspapers/magazines, reading books, playing chess, playing an instrument, listening to music, using internet/computer, and painting/drawing. Mental activity was categorized as (1) “intense” if the study participant participated in >3 activities/week; (2) “moderate” if the study participant participated in 2–3 activities/week; and (3) “low” if the participant participated in ≤1 activity/week [21,22].

Social activities included going to cinema, going to the theatre, attending concerts, visiting museums/art exhibitions, attending sport events, going to restaurants/pubs/cafes, playing bingo, dancing, attending religious activities, attending courses, travelling, attending social meetings, and doing voluntary work. It was categorized as (1) “intense” if the study participant reported participating in >1 activities/week; (2) “moderate” if the participant participated in 1 activity/week; and (3) “low” if the person participated in none of the activities in a week [21,22].

Based on the level of activities in the three dimensions, the total leisure activity score was assessed as “0” if at least two of the three activity dimensions were low and the third one was low/moderate; “1” if two of the dimensions were moderate and the third one was moderate/intense; and “2” if at least two of the dimensions were intense (other possible combinations were not seen in our cohort). An active lifestyle was defined as a leisure activity score of ≥1 (vs. 0 as inactive).

### 2.6. Statistical Methods

In order to correct the analyses for measurement error due to missing data, multiple imputation by chained Equation [23] was used to replace missing values in SFFQs and corresponding calorie intake estimates, which created 10 complete datasets. The imputed data were used in all analyses.

Baseline characteristics of the study population according to NPDP adherence levels were compared using chi-square (*χ*^2^) tests for categorical variables, quantile regression for continuous variables, and one-way Analysis of Variance (ANOVA) with Bonferroni correction for pairwise comparisons. Crude and multivariate mixed-effects linear regression models were used to examine the associations for (1) the NPDP adherence; (2) each of the physical, mental, and social activities; (3) joint NPDP adherence and each of the three activity dimensions; (4) joint NPDP and total leisure activity with rate of MMSE change over an average of 6 years. The fixed effects of the models included the interaction term between the exposure of interest (NPDP adherence levels and/or activities) and time. Random effects included the intercept and slope for time. Positive/negative β-coefficients for the (diet and/or activities × time) interactions (i.e., the slope) indicated lower/increased cognitive decline due to that specific exposure. In all analyses, individuals with low NPDP adherence and low activity levels were considered to be the reference group. All interactions between time and other covariates were also tested in a sensitivity analysis.

Further, hazard ratios (HR) and 95% confidence intervals (CI) for the risk of MMSE decline to ≤24 in relation to the NPDP and activity levels, both independently and in combination, were estimated by crude and multivariable parametric survival models. Follow-up time was censored at the date of examination in which MMSE decline to ≤24 was detected at death date or at the end of the 2nd follow-up, whichever occurred first. Additional sensitivity analyses included testing the effect of calorie misreporting and the effect of the imputation procedure on observed associations.

Age, sex, education, civil status, smoking status, and smoking duration among ever-smokers, body mass index (BMI), presence of a chronic disease (hypertension, stroke, ischemic heart disease, arrhythmias, heart failure, depression, diabetes, and cancer), dietary supplement use, *APOE* ɛ4 allele carriage, total calorie intake, and survival status were considered as established or proposed confounders in multiple imputation and data analyses. All statistical analyses were performed using Stata SE 14 (Stata Corporation, College Station, TX, USA). The statistical tests were two-sided and considered statistically significant at *p*-values < 0.05.

## 3. Results

### 3.1. Characteristics of the Study Population

Of the 2223 study participants, 39.2% (*n* = 871) were men, and 60.8% (*n* = 1352) were women. The mean age was 69.5 ± 8.6 years in men, and 71.3 ± 9.1 years in women. People with high NPDP adherence were younger and more likely to be married and be non-current smokers, have higher education, undergo intense physical activity, moderate-to-intense mental and social activities, and higher BMI; and were less likely to have vascular disorders, and had less deaths over follow-up than those with low NPDP adherence (Table 1). There were no significant differences in terms of sex, other chronic diseases, *APOE* ɛ4 allele carriage, dietary supplement use, and total energy intake among the three groups.

### 3.2. Associations between the NPDP and Leisure Activities with MMSE Change

In mixed-effects models, after multi-adjustment for potential confounders (including age, sex, education, civil status, total calorie intake, dietary vitamin/mineral supplement use, smoking status and duration, BMI, vascular disorders, other chronic diseases, *APOE* ɛ4 allele carriage, survival status, and diet or leisure activities where applicable), moderate or high adherence to NPDP, and moderate or intense physical, mental, and social activities were independently associated with lower cognitive decline compared to low diet adherence or low activity levels, correspondingly.

Because both moderate and high adherences to NPDP were related to less cognitive decline, the two categories were merged into one group as moderate-to-high adherence to NPDP. Likewise, both moderate and intense activity (including physical, mental and social dimensions) levels were similarly associated with lower rates of MMSE change, and were thus combined into a moderate-to-intense category for each activity in the following analyses.

Further, compared to those with a leisure activity score of “0” (inactive), both groups with scores of “1” and “2” showed significantly less cognitive decline, independent of dietary intake and other potential confounders, and were also grouped together as people with leisure activity scores “≥1” (active lifestyle) (Table 2).

In multi-adjusted parametric survival models, only moderate-to-high adherence to NPDP (HR: 0.50, 95% CI: 0.34 to 0.73) was independently associated with a reduced risk of MMSE decline to ≤24 over an average of 6 years compared to those with low diet adherence. Further, those with an active lifestyle had a significantly lower risk of MMSE decline to ≤24 relative to those with inactive lifestyle (HR: 0.65, 95% CI: 0.45 to 0.95) (Table S1).

### 3.3. Joint Effect of Healthy Diet and Leisure Activities on MMSE Decline

**Figure 1:**
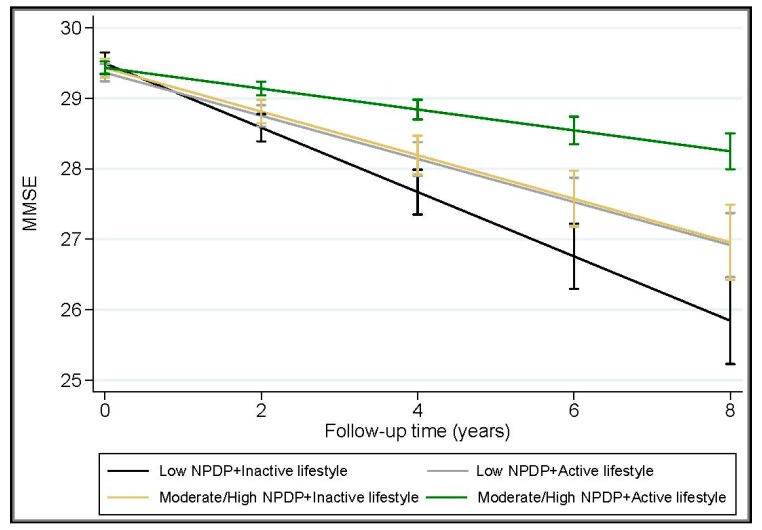
Joint effect of the Nordic Prudent Dietary Pattern (NPDP) and leisure activity on change in Mini-Mental State Examination (MMSE over an average of 6 years (range: 4 to 8 years) after adjustment for age, sex, education, civil status, total calorie intake, dietary vitamin/mineral supplement use, smoking status and duration, Body Mass Index (BMI), vascular disorders, diabetes, cancer, depression, *APOE* ɛ4 allele carriage, and survival status. Estimates are from the mixed-effects models (*n* = 2223). “Low NPDP” refers to low adherence to NPDP. “Moderate/High NPDP” refers to moderate-to-high adherence to NPDP. “Active lifestyle” refers to a leisure activity score ≥1. “Inactive lifestyle” refers to a leisure activity score = 0.

The joint exposure of NPDP adherence (moderate-to-high vs. low) with each of the physical, mental, and social activities (moderate-to-intense vs. low) was assessed by creating dummy variables and dividing the participants into four groups: (1) low NPDP adherence and low activity; (2) low NPDP adherence and moderate-to-intense activity; (3) moderate-to-high NPDP adherence and low activity; and (4) moderate-to-high NPDP adherence and moderate-to-intense activity. In multi-variable mixed-effects models, moderate-to-high adherence to NPDP together with moderate-to-intense physical (β: 0.34, 95% CI: 0.23 to 0.45), mental (β: 0.29, 95% CI: 0.21 to 0.37), or social (β: 0.27, 95% CI: 0.19 to 0.34) activities was associated with even lower rates of MMSE decline over an average of 6 years, compared to low NPDP adherence and low activity levels. An active lifestyle strengthened the protective effect of moderate-to-high adherence to NPDP on cognitive function by more than two times (β: 0.33, 95% CI: 0.24 to 0.42 vs. β: 0.16, 95% CI: 0.05 to 0.28) (Table 3 and Figure 1).

**Figure 2:**
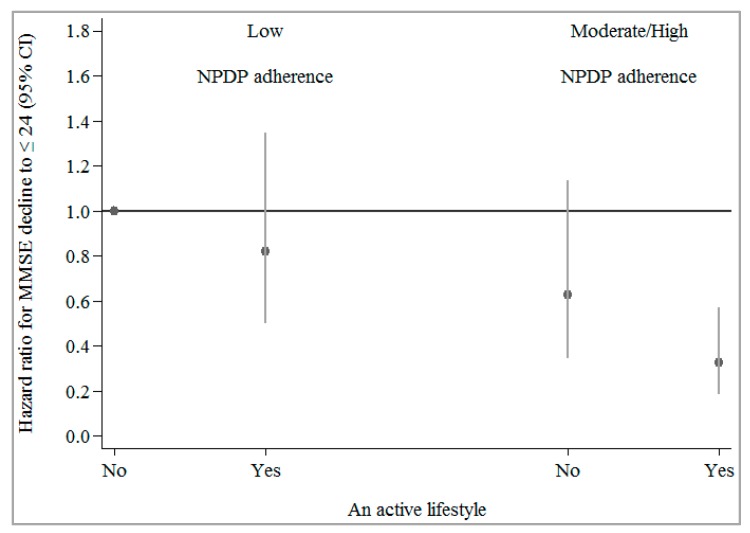
Joint effect of moderate-to-high (vs. low) adherence to the Nordic Prudent Dietary Pattern (NPDP) and an active lifestyle (yes vs. no) on MMSE decline to ≤24 over an average of 6 years (*n* = 1810). CI: Confidence Interval.

In multi-adjusted parametric survival models, moderate-to-high adherence to NPDP together with moderate-to-intense physical, mental, or social activity, was associated with an additional reduced risk of MMSE decline to ≤24 compared to the reference group (low adherence to NPDP and low activity) (Table 4). An active lifestyle may significantly strengthen the protective effect of moderate-to-high adherence to NPDP on MMSE decline to ≤24 (HR = 0.33 vs. HR = 0.63, representing a decrease in risk from 37% to 67%) (Figure 2).

Estimates were derived from the parametric survival models, adjusted for age, sex, education, civil status, total calorie intake, dietary vitamin/mineral supplement use, smoking status and duration (years), body mass index, vascular disorders, cancer, diabetes, depression, and *APOE* ɛ4 allele carriage. Total leisure activity score was assessed as “0” if at least two of the three activity dimensions (physical, mental, social) were low and the third one was low/moderate; “1” if two of the dimensions were moderate and the third one was moderate/intense; and “2” if at least two of the dimensions were intense. An active lifestyle (shown in the figure as “Yes”) was defined as a leisure activity score ≥1 (vs. 0 as inactive which is shown in figure as “No”).

### 3.4. Supplementary Analysis

In a sensitivity analysis, after adding the interaction terms between time and all covariates to the mixed-effects models, results of the associations between NPDP in combination with activity levels and MMSE change were slightly attenuated, but the direction of associations and significance remained. Further, in the analyses restricted to participants with complete data on the SFFQ (*n* = 815), the results for a combined effect of diet with activity levels on MMSE change and on MMSE decline to ≤24 remained mostly similar to those from the initial analyses. However, the strength of some associations was attenuated, most likely due to a loss of statistical power. All results from the supplementary analyses are available upon request.

## 4. Discussion

In this population-based prospective cohort study of cognitively normal Swedish older adults, we observed that moderate-to-intense physical, mental, or social activities may strengthen the association of moderate-to-high adherence to NPDP with lower cognitive decline. An active lifestyle may enlarge the protective effect of moderate-to-high adherence to NPDP on cognitive decline by more than two times and further lower the risk of MMSE decline to ≤24 by 30%. Our results suggest that an active lifestyle may significantly reinforce the beneficial effect of a healthy diet on cognitive function.

So far, intervention trials have shown positive associations of the Mediterranean and DASH diets with better cognitive outcomes [24,25]. Recently, the hybrid Mediterranean−DASH (MIND diet) was introduced as a protective dietary pattern against cognitive decline and Alzheimer’s disease [26]. However, the practicality and feasibility of following such dietary regimens in different populations with various food cultures, and their effects in combination with other lifestyle behaviors on cognition have been rarely investigated in the literature. We recently proposed the NPDP index [15] which may better predict the level of cognitive function preservation in Nordic elderly populations compared with other pre-defined healthful dietary patterns. Moderate-to-high adherence to NPDP is associated with less cognitive decline than the low adherence.

Leisure activities can be broadly divided into physical, social, and mental components. General consensus has emerged on the protective role of high leisure activities against dementia. Engaging in leisure activities has also been suggested to be a preventive lifestyle practice against cognitive decline. In a recent review of 17 prospective studies, higher participation in physical activity was associated with a 35% lower risk of decline in MMSE, relative to low participation in physical activity [27]. Although most cognitive training interventions have been implemented under laboratory or small-scale clinical conditions, there is some evidence showing that mentally-stimulating routine activities may delay or even decrease the risk of cognitive problems [28]. Very few systematic reviews of the evidence on social activities and cognitive decline are available [29], and it has been concluded that a socially integrated lifestyle in old age can be beneficial for maintaining cognitive health. Some studies have combined the three components of leisure activities to show that high participation leisure activities (moderate-to-intense in any two of the three components) is associated with a reduced dementia risk [30]. In line with these studies, our results indicated that moderate-to-intense participation in leisure activities, including physical, mental, and social components, is significantly associated with lower cognitive decline.

Following a healthy diet is only one of the factors constituting a healthy lifestyle. Another important one is leisure activity. More health-conscious individuals often follow not one, but several aspects of healthy behavior. Thus, investigations to find particular dimensions of lifestyle that are associated with disease risk are important. Nevertheless, many studies have focused on specific individual factors. One of the reasons for the failure to study diet in the past is the difficulty in summarizing dietary habits. This is particularly true in the neurological and dementia literature in which the methodological tool of dietary pattern assessment has been underused.

To our knowledge, there is scarce literature examining the combined effect of diet and leisure activity on cognitive function. Norton and colleagues evaluated different combinations of lifestyle factors in 2544 American individuals aged ≥65 and reported that different lifestyle behavior patterns are associated with different levels of risk for the incidence of dementia and Alzheimer’s disease (AD) [13]. Another study examining the combined effect of diet and physical activity on AD showed that high adherence to a Mediterranean-type diet in combination with a high level of participation in physical activity is associated with 35% lower risk of AD when compared to a low adherence to diet and low participation in physical activity [14]. In the present study, we found that moderate-to-intense participation in physical, mental, or social activities may strengthen the effect of moderate-to-high adherence to NPDP on cognitive function. An active lifestyle may enlarge the protective effect of moderate-to-high adherence to NPDP on cognitive decline by more than two times, and further lower the risk of MMSE decline to ≤24 by 30%. Our results suggest that an active lifestyle may significantly reinforce the beneficial effects of a healthy diet on cognitive function.

One of the well-known mechanisms through which lifestyle factors, especially a healthy diet and physical exercise, may indirectly contribute to brain protection is through the optimization of vascular health and reduction of the risk of atherosclerotic cerebrovascular diseases [31], and subsequently, vascular dementia. Diet and other lifestyle behaviors may exert their protective effects against cognitive aging through multiple and convergent pathways. For example, a diet enriched with docosahexaenoic acid (DHA; C22:6 *n*-3), other antioxidants [32] (e.g., vitamin E), flavonoids [33], and exercise involves similar molecular systems in the brain. The combination of these factors has an even greater impact on reducing oxidative stress and improving the synaptic plasticity mediated by the brain-derived neurotrophic factor (BDNF), and thus, enhances cognitive function [34,35]. Finally, lifestyle factors may affect both brain structure (brain reserve) and function (cognitive reserve), and slow down or mask cognitive decline through neuroprotective and/or compensatory mechanisms [36].

The main strengths of this study include its longitudinal design, relatively large sample size, low rate of dropouts at follow-up, corrections for measurement errors due to missing information, and assessment of the joint effect of lifestyle behaviors (dietary pattern and leisure activity) on cognitive decline. However, the limitations of the current study need to be acknowledged. First, this study used self-reported data on lifestyle factors. Thus, information bias as a systematic error due to inaccurate measurements of exposures is possible. In general, measurement errors in univariate exposure models may lead to the attenuation of regression coefficients or hazard ratios toward the null. Such errors in multivariate exposure settings may bias the estimates in any direction [37]. It is argued that individuals’ own perceptions or subjective experiences and interpretations of food intakes and activity participation may influence their reports, and thus, the observed associations. The presence of a recall or social desirability bias in these self-reported data cannot be ruled out. However, the findings were mostly in line with previous reports of lifestyle associations with cognitive decline. Second, data on single time-point (baseline) measures of diet and other lifestyle factors were used. The time-varying nature of these behaviors should be taken into account by using multiple measures of these factors over time. Following a stable lifestyle regimen in the long run, or at least over the study period, should be assured through repeated assessment of diet and other lifestyle behaviors. Third, shared characteristics of different leisure activities make it difficult to differentiate their impacts on cognition. However, simultaneous application of multiple healthful lifestyle practices may enhance their independent positive associations with lower cognitive decline. Finally, MMSE may not be able to capture mild degrees of cognitive decline, so, the use of a thorough cognitive battery is needed in future studies.

## 5. Conclusions

This study provides the first evidence that a healthy diet together with active engagement in physical, mental, and social leisure activities may additionally preserve cognitive function and even lessen the risk for MMSE decline to ≤24. These findings suggest that an active lifestyle may reinforce the beneficial effect of a healthy diet on cognitive function, and highlights the need for both healthy diet and active engagement in leisure activities in late life for better prevention of dementing disorders.

## Figures and Tables

**Table 1:** Baseline characteristics of the study population by tertile (low to high adherence) of the Nordic Prudent Dietary Pattern (NPDP) index (*n* = 2223).

Characteristics	Adherence to NPDP			*p*-Value *
	Low (*n* = 720)	Moderate (*n* = 779)	High (*n* = 724)	
NPDP score range	8 to 29	29 to 38	38 to 62	
Age, years	72.7 (66.3 to 81.3)	67.0 (60.8 to 78.3) *	66.2 (60.5 to 72.3) *	<0.001
Sex, women	437 (60.6)	474 (60.8)	442 (61.0)	0.985
Education				<0.001
University	193 (26.8)	283 (36.3)	337 (46.6)	
High school	330 (45.8)	344 (44.2)	300 (41.4)	
Elementary school	197 (27.4)	152 (19.5)	87 (12.0)	
Civil status				<0.001
Married	315 (43.7)	406 (52.0)	417 (57.6)	
Single	124 (17.3)	126 (16.2)	110 (15.1)	
Widow/Divorced	281 (39.0)	247 (31.8)	197 (27.3)	
Smoking				<0.001
Never	343 (47.7)	341 (43.7)	291 (40.1)	
Former	234 (32.5)	329 (42.3)	347 (47.9)	
Current	143 (19.8)	109 (14.0)	87 (12.0)	
Smoking (duration)	35 (19 to 45)	29 (15 to 42)	25 (15 to 38)	<0.001
Physical activity				<0.001
Low	165 (22.9)	133 (17.0)	92 (12.7)	
Moderate	428 (59.5)	431 (55.4)	390 (53.9)	
Intense	127 (17.6)	215 (27.6)	242 (33.4)	
Mental activity				<0.001
Low	364 (50.5)	278 (34.8)	180 (24.8)	
Moderate	247 (34.3)	322 (40.3)	281 (38.8)	
Intense	109 (15.2)	199 (24.9)	263 (36.4)	
Social activity				0.001
Low	466 (64.8)	503 (63.0)	393 (54.2)	
Moderate	162 (22.5)	190 (23.8)	204 (28.2)	
High	92 (12.7)	106 (13.2)	127 (17.6)	
BMI, kg/m^2^	25.0 (22.9 to 27.7)	25.5 * (23.2 to 27.9)	26.2 * (23.8 to 28.7)	<0.001
MMSE	29 (28 to 30)	29 (29 to 30)	29 (29 to 30)	1.000
Vascular disorders ^†^	646 (89.8)	704 (88.1)	614 (84.8) *	0.014
Other chronic diseases ^‡^	297 (41.3)	328 (41.1)	267 (36.9)	0.168
Any *APOE* ɛ4 allele carriage	217 (30.2)	214 (26.8)	222 (30.6)	0.279
Dietary supplement use	211 (29.3)	216 (27.0)	197 (27.1)	0.550
Total energy intake, kcal/day	1620.0 (1289.0 to 2035.5)	1571.1 (1235.3 to 1937.9)	1628.8 (1324.4 to 1968.7)	0.984
Deaths at follow-ups	192 (26.7)	136 (17.4)	71 (9.8)	<0.001

Abbreviations: NPDP, Nordic Prudent Dietary Pattern; BMI, body mass index; MMSE, Mini-Mental State Examination; *APOE*, apolipoprotein E.; Values are number (%) for categorical variables, and median (interquartile range) for continuous variables. Chi-square test for categorical variables, and quantile regression for continuous variables. * Pairwise mean comparisons using Bonferroni correction: *p* < 0.05 (reference group was low adherence to NPDP). ^†^ Vascular disorders include hypertension, hypercholesterolemia, stroke, heart diseases (including coronary heart disease, arrhythmia and heart failure). ^‡^ Diabetes, cancer, and depression.

**Table 2:** β-coefficients with 95% confidence intervals (CI) for the associations of the Nordic Prudent Dietary Pattern (NPDP) and leisure activities with the rate of change in MMSE over an average of 6 years (*n* = 2223).

Lifestyle Factors	*n*	β (95% CI)			
		Model 1 *	*p*-Value	Model 2 ^†^	*p*-Value
**Adherence to NPDP**					
Low	720	Reference		Reference	
Moderate-to-high	1503	0.19 (0.13 to 0.25)	<0.001	0.19 (0.14 to 0.24)	<0.001
Moderate	779	0.14 (0.08 to 0.21)	<0.001	0.14 (0.08 to 0.20)	<0.001
High	724	0.24 (0.18 to 0.31)	<0.001	0.24 (0.18 to 0.30)	<0.001
*p*-value for trend		0.12 (0.09 to 0.15)	<0.001	0.12 (0.09 to 0.15)	<0.001
**Physical Activity**					
Low	390	Reference		Reference	
Moderate-to-intense	1833	0.13 (0.06 to 0.20)	<0.001	0.13 (0.07 to 0.20)	<0.001
Moderate	1249	0.11 (0.03 to 0.18)	0.005	0.11 (0.04 to 0.18)	0.003
Intense	584	0.18 (0.10 to 0.26)	<0.001	0.19 (0.11 to 0.26)	<0.001
*p*-value for trend		0.09 (0.05 to 0.13)	<0.001	0.09 (0.05 to 0.13)	<0.001
**Mental Activity**					
Low	815	Reference		Reference	
Moderate-to-intense	1408	0.15 (0.09 to 0.22)	<0.001	0.15 (0.09 to 0.21)	<0.001
Moderate	842	0.11 (0.03 to 0.18)	0.006	0.10 (0.03 to 0.18)	0.006
Intense	566	0.22 (0.15 to 0.29)	<0.001	0.22 (0.15 to 0.29)	<0.001
*p*-value for trend		0.11 (0.07 to 0.14)	<0.001	0.11 (0.07 to 0.14)	<0.001
**Social Activity**					
Low	1349	Reference		Reference	
Moderate-to-intense	874	0.09 (0.04 to 0.15)	0.001	0.09 (0.04 to 0.14)	<0.001
Moderate	552	0.08 (0.01 to 0.14)	0.019	0.08 (0.02 to 0.14)	0.011
Intense	322	0.12 (0.04 to 0.20)	0.003	0.12 (0.04 to 0.19)	0.003
*p*-value for trend		0.06 (0.03 to 0.10)	0.001	0.06 (0.03 to 0.10)	<0.001
**Leisure Activity Score**					
0 (Inactive)	614	Reference		Reference	
≥1 (Active)	1609	0.20 (0.14 to 0.26)	<0.001	0.20 (0.14 to 0.26)	<0.001
1	1269	0.18 (0.12 to 0.25)	<0.000	0.18 (0.12 to 0.25)	<0.001
2	340	0.26 (0.18 to 0.34)	<0.001	0.26 (0.18 to 0.34)	<0.001
*p*-value for trend		0.14 (0.10 to 0.18)	<0.001	0.14 (0.10 to 0.18)	<0.001

β-coefficients (95% Confidence Interval (CI)) are interactions with time from the mixed-effects models. Positive coefficients refer to less decline in MMSE compared to the reference group. * Model 1: crude. ^†^ Model 2: adjusted for age, sex, education, civil status, total calorie intake, dietary vitamin/mineral supplement use, smoking status and duration (years), body mass index, vascular disorders, cancer, diabetes, depression, *APOE* ɛ4 allele carriage, survival status, and other lifestyle factors than the main exposure in each model.

**Table 3:** β-coefficients (95% CI) of the joint effects of leisure activities (including physical, mental, and social dimensions) and adherence to the Nordic Prudent Dietary Pattern (NPDP) on MMSE changes over an average of 6 years (*n* = 2223).

Joint Exposure		*n*	Model 1 *	*p*-Value	Model 2 ^†^	*p*-Value
Physical Activity	NPDP Adherence		β (95% CI)		β (95% CI)	
Low	Low	165	Reference		Reference	
Moderate/intense	Low	554	0.18 (0.05 to 0.31)	0.007	0.18 (0.05 to 0.30)	0.005
Low	Moderate/high	224	0.28 (0.12 to 0.43)	0.001	0.27 (0.12 to 0.42)	0.001
Moderate/intense	Moderate/high	1280	0.34 (0.23 to 0.46)	<0.001	0.34 (0.23 to 0.45)	<0.001
*p*-value for trend			0.09 (0.07 to 0.12)	<0.001	0.09 (0.07 to 0.12)	<0.001
**Mental Activity**	**NPDP Adherence**					
Low	Low	362	Reference		Reference	
Moderate/intense	Low	357	0.14 (0.04 to 0.24)	0.006	0.13 (0.02 to 0.24)	0.016
Low	Moderate/high	452	0.18 (0.09 to 0.27)	<0.001	0.18 (0.09 to 0.26)	<0.001
Moderate/intense	Moderate/high	1052	0.30 (0.22 to 0.37)	<0.001	0.29 (0.21 to 0.37)	<0.001
*p*-value for trend			0.09 (0.07 to 0.12)	<0.001	0.09 (0.07 to 0.12)	<0.001
**Social Activity**	**NPDP Adherence**					
Low	Low	467	Reference		Reference	
Moderate/intense	Low	252	0.10 (−0.02 to 0.21)	0.097	0.10 (−0.01 to 0.20)	0.066
Low	Moderate/high	883	0.19 (0.12 to 0.27)	<0.001	0.19 (0.12 to 0.27)	<0.001
Moderate/intense	Moderate/high	621	0.27 (0.19 to 0.34)	<0.001	0.27 (0.19 to 0.34)	<0.001
*p*-value for trend			0.09 (0.07 to 0.11)	<0.001	0.09 (0.07 to 0.11)	<0.001
**Leisure Activity Score**	**NPDP Adherence**					
0 (Inactive)	Low	275	Reference		Reference	
≥1 (Active)	Low	445	0.17 (0.06 to 0.29)	0.003	0.17 (0.06 to 0.28)	0.003
0 (Inactive)	Moderate/high	339	0.16 (0.05 to 0.28)	0.007	0.16 (0.05 to 0.28)	0.006
≥1 (Active)	Moderate/high	1164	0.33 (0.24 to 0.42)	<0.001	0.33 (0.24 to 0.42)	<0.001
*p*-value for trend			0.07 (0.05 to 0.08)	<0.001	0.07 (0.05 to 0.08)	<0.001

β-coefficients (95% CI) are interactions with time from the mixed-effects models. Positive coefficients refer to less decline in MMSE, and negative coefficients indicate more decline in MMSE compared to the reference group. * Model 1: crude. ^†^ Model 2: adjusted for age, sex, education, civil status, total calorie intake, dietary vitamin/mineral supplement use, smoking status and duration, body mass index, vascular disorders, diabetes, cancer, depression, *APOE* ɛ4 allele carriage, survival status, and physical, mental and social activities, where applicable.

**Table 4:** Hazard ratios and 95% confidence intervals (CI) for the joint effect of leisure activities (including physical, mental, and social dimensions) and adherence to the Nordic Prudent Dietary Pattern (NPDP) on MMSE decline to ≤24 over an average of 6 years (*n* = 1810).

Joint Exposure		*n*	Model 1 *	*p*-Value	Model 2 ^†^	*p*-Value
Physical Activity	NPDP Adherence		HR (95% CI)		HR (95% CI)	
Low	Low	165	Reference		Reference	
Moderate/intense	Low	554	0.62 (0.38 to 1.02)	0.058	0.80 (0.48 to 1.33)	0.394
Low	Moderate/high	224	0.28 (0.12 to 0.63)	0.002	0.55 (0.24 to 1.27)	0.160
Moderate/intense	Moderate/high	1280	0.16 (0.09 to 0.27)	<0.001	0.39 (0.22 to 0.67)	0.001
*p*-value for trend			0.53 (0.45 to 0.62)	<0.001	0.72 (0.61 to 0.85)	<0.001
**Mental Activity**	**NPDP Adherence**					
Low	Low	362	Reference		Reference	
Moderate/intense	Low	357	0.73 (0.46 to 1.17)	0.189	1.04 (0.63 to 1.71)	0.884
Low	Moderate/high	452	0.37 (0.22 to 0.62)	<0.001	0.63 (0.37 to 1.09)	0.096
Moderate/intense	Moderate/high	1052	0.16 (0.09 to 0.27)	<0.001	0.40 (0.22 to 0.73)	0.003
*p*-value for trend			0.55 (0.47 to 0.64)	<0.001	0.74 (0.62 to 0.87)	<0.001
**Social Activity**	**NPDP Adherence**					
Low	Low	467	Reference		Reference	
Moderate/intense	Low	252	0.69 (0.41 to 1.17)	0.170	0.83 (0.49 to 1.41)	0.498
Low	Moderate/high	883	0.31 (0.21 to 0.48)	<0.001	0.57 (0.37 to 0.88)	0.011
Moderate/intense	Moderate/high	621	0.11 (0.06 to 0.22)	<0.001	0.28 (0.14 to 0.57)	<0.001
*p*-value for trend			0.52 (0.44 to 0.61)	<0.001	0.70 (0.59 to 0.83)	<0.001

Hazard ratios (95% CI) are from the parametric survival models. * Model 1: crude. ^†^ Model 2: adjusted for age, sex, education, civil status, total calorie intake, dietary vitamin/mineral supplement use, smoking status and duration (years), body mass index, vascular disorders, cancer, diabetes, depression, *APOE* ɛ4 allele carriage, and lifestyle factors other than the main exposure in each model.

## Supplementary Materials

The following are available online at http://www.mdpi.com/2072-6643/10/9/1297/s1, Figure S1: Flowchart of the study population in the Swedish National Study on Aging and Care in Kungsholmen (SNAC-K), Stockholm, Sweden. MMSE, Mini-Mental State Examination; SFFQ, semi-quantitative food frequency questionnaire, Table S1: Hazard ratios (HR) and 95% confidence intervals (CI) for the associations of the Nordic Prudent Dietary Pattern (NPDP) and leisure activities with the MMSE decline to ≤24 over an average of 6 years (*n* = 1810).
