# Supplementary material for: An Active Lifestyle Reinforces the Effect of a Healthy Diet on Cognitive Function: A Population-Based Longitudinal Study
Source: Nutrients. 2018 Sep 13;10(9):1297. doi: 10.3390/nu10091297 (PMC6163666; doi:10.3390/nu10091297)
Supplement: Supplementary file 1 [file nutrients-10-01297-s001.pdf]

Table S1. Hazard ratios (HR) and 95% confidence intervals (CI) for the associations of the Nordic Prudent Dietary Pattern (NPDP) and leisure activities with the MMSE decline to ≤24 over the average of 6 years (n = 1810).

Lifestyle factors	n	HR (95% CI)			
		Model 1*	*P*	Model 2†	*P*
**Adherence to NPDP**					
Low	720	Reference		Reference	
Moderate-to-high	1503	0.25 (0.17 to 0.36)	<0.001	0.50 (0.34 to 0.73)	<0.001
Moderate	779	0.40 (0.27 to 0.60)	<0.001	0.64 (0.42 to 0.96)	0.032
High	724	0.10 (0.05 to 0.21)	<0.001	0.25 (0.12 to 0.53)	<0.001
P for trend		0.35 (0.26 to 0.46)	<0.001	0.55 (0.42 to 0.73)	<0.001
**Physical activity**					
Low	390	Reference		Reference	
Moderate-to-intense	1833	0.52 (0.35 to 0.77)	0.001	0.75 (0.50 to 1.14)	0.180
Moderate	1249	0.61 (0.40 to 0.91)	0.015	0.75 (0.49 to 1.15)	0.194
Intense	584	0.36 (0.21 to 0.60)	<0.001	0.75 (0.43 to 1.32)	0.324
P for trend		0.60 (0.46 to 0.77)	<0.001	0.85 (0.64 to 1.14)	0.279
**Mental activity**					
Low	815	Reference		Reference	
Moderate-to-intense	1408	0.46 (0.31 to 0.66)	<0.001	0.90 (0.59 to 1.37)	0.619
Moderate	842	0.65 (0.43 to 0.97)	0.034	1.03 (0.67 to 1.60)	0.874
Intense	566	0.20 (0.11 to 0.38)	<0.001	0.54 (0.27 to 1.07)	0.078
P for trend		0.51 (0.39 to 0.66)	<0.001	0.82 (0.61 to 1.10)	0.194
**Social activity**					
Low	1349	Reference		Reference	
Moderate-to-intense	874	0.48 (0.32 to 0.72)	<0.001	0.68 (0.45 to 1.04)	0.075
Moderate	552	0.47 (0.29 to 0.76)	0.002	0.67 (0.41 to 1.09)	0.109
Intense	322	0.50 (0.27 to 0.93)	0.028	0.71 (0.38 to 1.32)	0.275
P for trend		0.63 (0.47 to 0.85)	0.002	0.79 (0.59 to 1.06)	0.121
**Leisure activity score‡**					
0 (Inactive)	614	Reference		Reference	

≥1 (Active)	1609	0.39 (0.27 to 0.56)	<0.001	0.65 (0.45 to 0.95)	0.027
1	1269	0.44 (0.31 to 0.64)	<0.001	0.70 (0.48 to 1.03)	0.073
2	340	0.21 (0.10 to 0.43)	<0.001	0.42 (0.20 to 0.89)	0.024
P for trend		0.45 (0.33 to 0.60)	<0.001	0.68 (0.50 to 0.91)	0.011

Hazard ratios (95% CI) are from the parametric survival models. * Model 1: crude. † Model 2: adjusted for age, sex, education, civil status, total calorie intake, dietary vitamin/mineral supplement use, smoking, body mass index, vascular disorders, cancer, diabetes, depression, *APOE* ε4 allele carriage, and physical, mental and social activities when applicable. ‡ Total leisure activity score was assessed as "0" if at least two of the three activity dimensions (physical, mental, social) were low, and the third one was low/moderate, "1" if two of the dimensions were moderate, and the third one was moderate/intense, and "2" if at least two of the dimensions were intense. An active lifestyle was defined if the leisure activity score was ≥1 (vs. 0 as inactive).

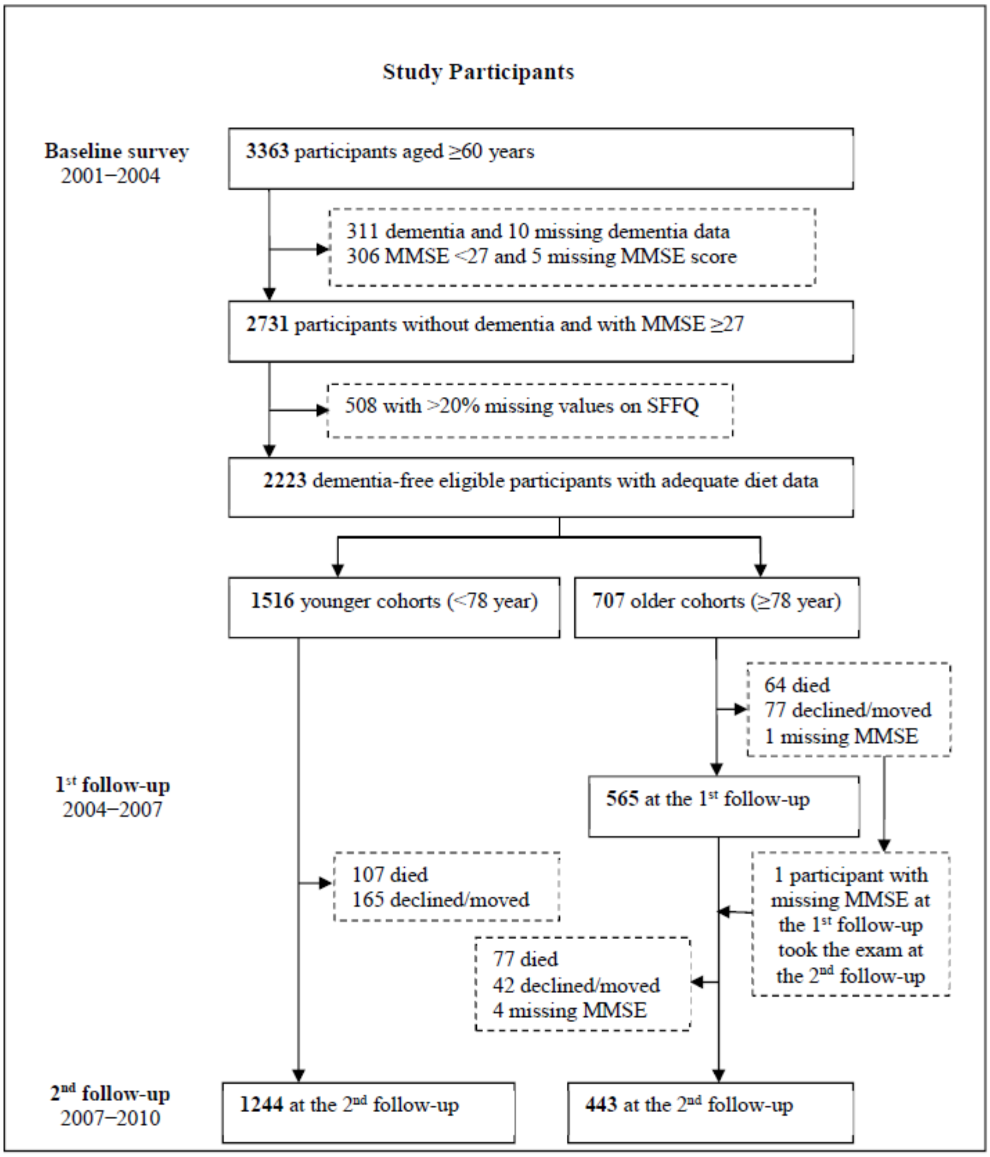

Figure S1. Flowchart of the study population in the Swedish National Study on Aging and Care in Kungsholmen (SNAC-K), Stockholm, Sweden. MMSE, Mini-Mental State Examination; SFFQ, semi-quantitative food frequency questionnaire.
